# Gouty Tophi in the Penis: A Case Report and Review of the Literature

**DOI:** 10.1155/2012/594905

**Published:** 2012-07-16

**Authors:** José Francisco Flores Martín, Fernando Vázquez Alonso, Ignacio Puche Sanz, Raquel Berrio Campos, Miguel Angel Campaña Gutierrez, José Manuel Cózar Olmo

**Affiliations:** Servicio de Urología, Hospital Universitário, Virgen de las Nieves, 18014 Granada, Spain

## Abstract

Gout is a metabolic disease characterized by hyperuricemia and the deposition of monosodium urate crystals in different anatomical locations. We report the case of a 61-year-old man who received consultation for gouty tophi in the penis, which is an unusual location for this type of pathology, that was resolved with the surgical removal of the tophi. We provide a review on gout and its treatment as well as other locations where atypical gouty tophi have been described.

## 1. Introduction

Gout is a systemic disorder characterized by recurrent bouts of arthritis and hyperuricemia, with the deposition of monosodium urate crystals in the synovial spaces and other extra-articular locations. Gout is manifested by the presence of subcutaneous lesions called gouty tophi.

The most common sites of tophi include the helix or antihelix, the olecranon bursae and the patellar periarticular ligaments, and the Achilles tendon as well as the dermis and subcutaneous tissue of the hands, feet, and elbows. The subcutaneous lesions are variable in number, size, and shape, and they feature yellowish-white color with firm, smooth or ulcerated appearance and are, in most cases, highlighted on the skin [1, 2]. 

We report the case of a patient with penis tophi, which represents an exceptional location for this condition.

## 2. Case Presentation

A 61-year-old male presented with a history of hypertension and years of hyperuricemia and gouty arthropathy. He consulted for 4 small lesions present on a raised, irregular, erythematous penis that caused him pain during erection. The lesions were not adherent to deep planes, and the largest lesion was 2.5 cm, suggestive of gouty tophi.

The patient also exhibited similar lesions of various sizes on his hands, feet, and elbows, which did not cause symptoms. He was treated with allopurinol and NSAIDs, but despite treatment, the lesions did not disappear.

The patient's analytical findings showed only a mild renal insufficiency (creatinine of 1.5 mg/dL) and hyperuricemia of 9 mg/dL.

**Figure 1:**
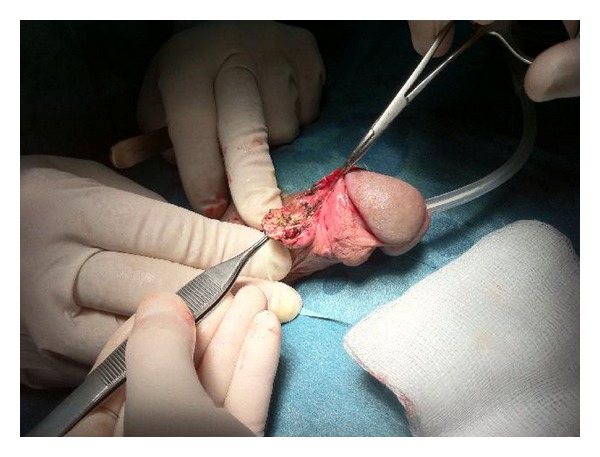
Surgical excision of the tophi in the penis.

**Figure 2:**
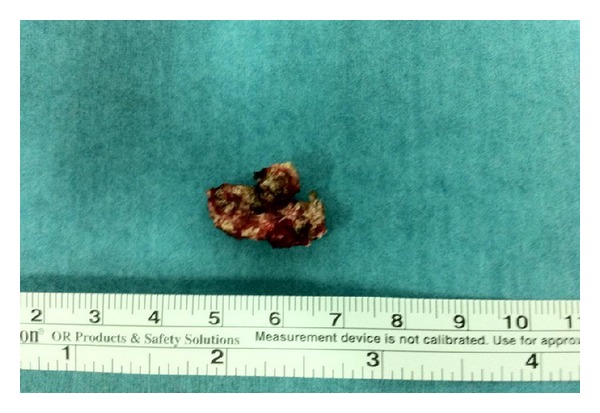
Tophi gouty of 2,5 cm after excision.

We performed a surgical excision of the tophi from the penis, without incident (Figures 1 and 2). The excised fragments were sent to pathology, confirming the gouty tophi (based on monosodium urate levels). The patient was asymptomatic for 9 months after surgery. 

## 3. Discussion

Gout is a metabolic disease, characterized by the deposition of monosodium urate crystals in the interior of the joints and other tissues. This condition occurs in patients with serum concentrations of uric acid greater than 7 mg/dL, either due to an increase in production or a decrease in excretion [3].

Gout is a disease more commonly found in men than in women [4] (i.e., 9 male cases for each case in a woman) and usually occurs during the 5th and 6th decades of life, as observed in this case (a 61-year-old male) [5].

The disease can manifest in four forms: asymptomatic hyperuricemia, gouty arthritis, intercritical gout, and chronic tophaceous gout, as observed in this case. In the chronic phase of the tophaceous gout, the tophi appear and consist of a mass of monosodium urate crystals. These crystals are surrounded by cells that typically characterize an inflammatory response, including immature fibroblasts, lymphocytes, plasma cells, macrophages, and foreign body giant cells [6].

Gouty arthritis is evident when tissues have been exposed to hyperuricemic fluids for years. The initial clinical presentation is often due to the involvement of the first metatarsophalangeal joint, followed by the ankles, knees, wrists, and the interphalangeal hand and shoulder. Gouty tophi have been documented to occur after 5 years of the development of arthritis and are usually visible under the skin, joints, and limbs. They are commonly found in the olecranon bursa, the infrapatellar and Achillestendons, in the subcutaneous tissue of the extensor surfaces of forearms, on the joints, and occasionally in the helix handset. The intradermal tophi are less frequently located on the palms and fingertips [7].

Gouty tophi have been reported in other unusual locations, such as the arytenoid cartilage, vocal cords, laryngeal tophi, myocardium, cardiac conduction system, mitral and aortic valves, eyes, and spinal cord [6].

The primary medical treatment for chronic tophaceous gout includes alterations in lifestyle (i.e., weight reduction, low-purine diet, high fluid intake, reduced alcohol consumption, and withdrawal of diuretics). If the hyperuricemia cannot be corrected by these methods, we will have to use drugs. As there are several types of drugs to consider, we should use the characteristics of our patients to guide treatment selection. Probenecid is a uricosuric drug that can be used in patients with good kidney function, who excrete some uric acid. In patients with renal failure, and who are taking diuretics, the choice medication would be another type of uricosuric and/or allopurinol [8]. The use of uricosurics has permanently decreased tophaceous gout and the associated arthritic changes by almost 50% when compared with their disuse [6]. In the present case, our patient was treated with allopurinol; however, despite this treatment, the tophi were not controlled.

The indication for a surgical intervention was guided by the poor response to treatment, as these methods are usually effective in the vast majority of cases. Surgical treatment may be indicated depending on the extent of the patient's problem and the functional impairment due to the compression of structures [9].

The appearance of the gouty tophi in the penis represents an exceptional location. Given its rarity, there is no elective treatment. As in our case, given the failure of medical treatment, one may opt for surgery.

The appearance of the gouty tophi is an expression of the urate deposits that commonly occurs in patients who suffer from this disease over the long term. If medical treatments fail and the tophi become symptomatic, one can proceed with their surgical removal.
